# Spatial Distribution and Long-Term Alterations of Peripheral Nerve Lesions in Schwannomatosis

**DOI:** 10.3390/diagnostics12040780

**Published:** 2022-03-23

**Authors:** Tim Godel, Philipp Bäumer, Said Farschtschi, Barbara Hofstadler, Sabine Heiland, Mathias Gelderblom, Martin Bendszus, Victor-Felix Mautner

**Affiliations:** 1 Department of Neuroradiology, Neurological University Clinic, Heidelberg University Hospital, Im Neuenheimer Feld 400, 69120 Heidelberg, Germany; p.baeumer@dialog-aoe.de (P.B.); barbara.hofstadler@outlook.com (B.H.); sabine.heiland@med.uni-heidelberg.de (S.H.); martin.bendszus@med.uni-heidelberg.de (M.B.); 2 Center for Radiology dia.log, Vinzenz-von-Paul Str. 8, 84503 Altotting, Germany; 3 Department of Neurology, University Medical Center Hamburg-Eppendorf, Martinistraße 52, 20246 Hamburg, Germany; s.farschtschi@uke.de (S.F.); mgelderb@uke.de (M.G.); v.mautner@uke.de (V.-F.M.)

**Keywords:** magnetic resonance neurography, schwannomatosis, small fiber neuropathy, peripheral nerve lesions

## Abstract

Purpose To examine the spatial distribution and long-term alterations of peripheral nerve lesions in patients with schwannomatosis by in vivo high-resolution magnetic resonance neurography (MRN). Methods In this prospective study, the lumbosacral plexus as well as the right sciatic, tibial, and peroneal nerves were examined in 15 patients diagnosed with schwannomatosis by a standardized MRN protocol at 3 Tesla. Micro-, intermediate- and macrolesions were assessed according to their number, diameter and spatial distribution. Moreover, in nine patients, peripheral nerve lesions were compared to follow-up examinations after 39 to 71 months. Results In comparison to intermediate and macrolesions, microlesions were the predominant lesion entity at the level of the proximal (*p* < 0.001), mid- (*p* < 0.001), and distal thigh (*p* < 0.01). Compared to the proximal calf level, the lesion number was increased at the proximal (*p* < 0.05), mid- (*p* < 0.01), and distal thigh level (*p* < 0.01), while between the different thigh levels, no differences in lesion numbers were found. In the follow-up examinations, the lesion number was unchanged for micro-, intermediate and macrolesions. The diameter of lesions in the follow-up examination was decreased for microlesions (*p* < 0.01), not different for intermediate lesions, and increased for macrolesions (*p* < 0.01). Conclusion Microlesions represent the predominant type of peripheral nerve lesion in schwannomatosis and show a rather consistent distribution pattern in long-term follow-up. In contrast to the accumulation of nerve lesions, primarily in the distal nerve segments in NF2, the lesion numbers in schwannomatosis peak at the mid-thigh level. Towards more distal portions, the lesion number markedly decreases, which is considered as a general feature of other types of small fiber neuropathy.

## 1. Introduction

Schwannomatosis, also referred to as the third type of neurofibromatosis, is a hereditary tumor predisposition syndrome. Its clinical symptoms are primarily characterized by the development of small fiber neuropathy (SFN), affecting predominantly small unmyelinated C fibers, causing severe acroparesthesia and chronic pain [1]. 

Although the clinical symptoms and genetic background differ greatly from Neurofibromatosis Type 2 (NF2), both syndromes also share major pathomorphological and imaging features. First, schwannomas are the predominant tumor entity, with identical histological and imaging features. Second, both syndromes share polyneuropathy (PNP) as a major clinical finding and a compressive impact of schwannomas on adjacent peripheral nerves was first considered as the major cause of these neuropathic symptoms. Third, recent studies found that intrafascicular microlesions, rather than mass schwannomas, play a critical role in the development of PNP with a close correlation between the number of nerve lesions, severity of clinical symptoms, and electrophysiological measures [2].

Despite the considerable pathomorphological overlap, clinical features differ greatly between NF2 and schwannomatosis. While PNP in NF2 patients is characterized by mixed, axonal and demyelinating nerve fiber damage and consecutive motor and sensory symptoms [3], the most common and predominant symptoms of schwannomatosis are severe acroparesthesia and chronic pain [4]. While similarities between nerve lesions in both conditions and their critical role in the development of PNP have already been thoroughly described, the distribution of lesions and their alterations over time in schwannomatosis are so far unknown. Thus, this study examined the spatial distribution of micro-, intermediate- and macrolesions within the nerve segments of the lower extremities in schwannomatosis patients and their alterations in long-term follow-up. 

## 2. Subjects and Methods

### 2.1. Clinical and Demographic Patient Data

This study was performed in accordance with the Declaration of Helsinki and approved by the institutional ethics board (S398-2012), and written informed consent was obtained from all patients. All patients were in neurological long-term care at the Department of Neurology, University Medical Center Hamburg-Eppendorf. Clinical examination, neurological and genetic testing were performed by a neurologist with more than 35 years of experience with NF2 and schwannomatosis (V.-F.M.). Initial and follow-up MRN examinations were performed between 08/2010 and 5/2019. Overall, we included 15 patients with schwannomatosis (nine males, six females, Table 1) and comprehensive baseline evaluation, of which nine patients were examined additionally by clinical and MRN follow-up examination. The clinical diagnosis was based on the revised consensus criteria for schwannomatosis. All patients underwent genetic analysis for the NF2, SMARCB1, and LZTR1 genes in the blood and, if available, in tumor tissue, as reported previously. 

### 2.2. Electrophysiological Testing and Skin Biopsies

Nerve conduction studies, electromyography and determination of intraepidermal nerve fiber density (IENFD) were performed in accordance with previous studies [2,5]. The results are given in Table 1.

### 2.3. Imaging Protocol

All MRN examinations were performed on three Tesla Magnetic Resonance scanners (Magnetom VERIO, Magnetom SKYRA and Magnetom TIM TRIO, Siemens Healthineers, Erlangen, Germany). A 15-channel receive/transmit spine coil and an 8-channel receive body flex coil (Siemens Healthcare) were used for imaging of the lumbosacral plexus, and a 15-channel transmit/receive knee-coil (Invivo, Gainesville, FL, USA) was used for imaging the lower extremity. Initially, all 15 patients underwent large-coverage high-resolution MRN of the lumbosacral plexus and the lower extremities, including:

1. A 3D T2-weighted sampling-perfection-with-application-optimized-contrasts-using-different-flip-angle-evolution (SPACE) short-tau-inversion-recovery (STIR) sequence of the lumbosacral plexus with the following parameters: effective echo time 68 ms, repetition time/echo time 3000/208 ms, inversion time 210 ms, field of view 305 × 305 mm^2^, slice thickness 0.95 mm, matrix size 320 × 320 × 104, no gap, voxel size 0.95 × 0.95 × 0.95 mm^3^, acquisition time 8:35 min, covering the lumbosacral spine from the second lumbar vertebra to the coccyx with 3D reformations in axial, sagittal and coronal orientations.
2. A T2-weighted, turbo-spin-echo sequence of the right thigh, knee and calf level in an axial orientation, using the following parameters: echo time 59 ms, repetition time 8.470 ms, spectral fat saturation, matrix 512 × 512, field of view 160 × 160 mm, voxel size 0.3 × 0.3 × 3.5 mm^3^, interslice gap 0.35 mm, 45 slices for each slab, acquisition time 7:56 min per slab.

Follow-up examinations in nine patients were performed using a reduced imaging protocol including a T2-weighted, turbo-spin-echo sequence of the right thigh, knee and calf level, using identical parameters, as described above. 

### 2.4. Imaging Analysis 

Image post-processing was performed by a board-certified radiologist with 8 years of experience in musculoskeletal and peripheral nerve imaging (T.G.). Assessment of fascicular T2-signal was performed in the initial and follow-up examinations within the right-sided sciatic, tibial, and peroneal nerves slice-by-slice from proximal to distal. Additionally, the right-sided nerve roots L5 and S1 were analyzed in the segment between the dorsal root ganglion (DRG) and the infrapiriform foramen in the initial examination. To address the spatial distribution of nerve lesions, cumulative lesion count was plotted against 60 consecutive slice positions along the course of the right sciatic, tibial and peroneal nerve. To ensure comparability between patients, the middle portion of the anterior cruciate ligament was defined as slice position 0. More proximal slices were assigned positive, and more distal slices negative slice positions. Therefore, the proximal thigh ranged from slice position 45 to 30, the middle thigh from 30 to 15, the distal thigh from 15 to 0, and the proximal calf from 0 to −15. 

A peripheral nerve lesion was identified by an alteration of fascicular architecture in combination with an increased T2 signal of the affected fascicle and classified into one of the following categories by their largest cross-sectional diameter [2]:

1. “microlesion” if the largest diameter of the lesion was <2 mm.
2. “intermediate lesion” if the largest diameter of the lesion was between 2 and 5 mm.
3. “macrolesion” if the largest diameter of the lesion was >5 mm.

### 2.5. Statistical Analysis

Statistical analyses were performed using GraphPad Prism 7.0 (GraphPad Software, La Jolla, CA, USA). The total number of peripheral nerve lesions in each of the categories listed above was counted for each level and slice position and tested for significance using a one-way analysis of variance (ANOVA) with Bonferroni correction. Moreover, lesion number and lesion diameter were determined in the initial and follow-up examination and tested for significant differences using paired, two-sided Student’s *t*-tests. Results are documented as mean values ± standard deviation, and *p*-values of <0.05 were considered significant. 

## 3. Results

**Figure 1:**
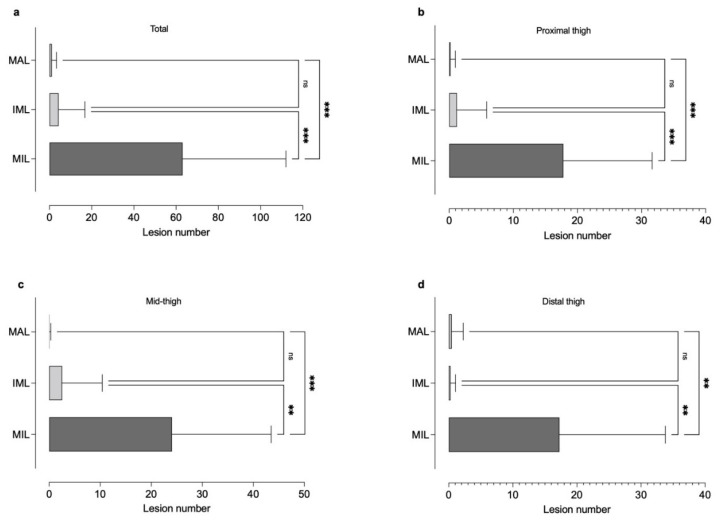
Different types of nerve lesion and their appearance (**a**) at the proximal thigh (**b**), mid-thigh (**b**), distal thigh (**c**), and calf (**d**) level. While peripheral nerve microlesions (MIL) were the predominant lesion entity at all thigh levels, there was no difference between the number of intermediate (IML) and macrolesions (MAL) (Ns, non-significant, ** *p* < 0.01, *** *p* < 0.001).

Peripheral nerve lesions were quantitatively assessed for a total of 15 patients diagnosed with schwannomatosis. Electrophysiological testing and determination of IENFD were partially published in a previous study [4,5] and are given in Table 1. In the initial examination, a total of 946 (63.07 ± 48.95) micro-, 65 (4.33 ± 12.48) intermediate, and 17 (1.13 ± 2.23) macrolesions were found along the course of the right sided L5/S1 root, the sciatic, tibial and peroneal nerve. Compared to the number of intermediate and macrolesions, the number of microlesions was higher at the proximal thigh (17.80 ± 13.86 vs. 1.20 ± 4.65 vs. 0.20 ± 0.77, Bonferroni corrected *p* < 0.001), the mid-thigh (24.07 ± 19.43 vs. 2.53 ± 7.87 vs. 0.07 ± 0.26, Bonferroni corrected *p* < 0.001), and the distal thigh (17.27 ± 16.52 vs. 0.27 ± 0.80 vs. 0.47 ± 1.81, Bonferroni corrected *p* < 0.01). At the pelvic (0.33 ± 0.90 vs. 0.13 ± 0.52 vs. 0.07 ± 0.26) and proximal calf level (3.60 ± 7.01 vs. 0.20 ± 0.56 vs. 0.20 ± 0.56) no differences between the lesion count in the different categories were found (Figure 1). 

**Figure 2:**
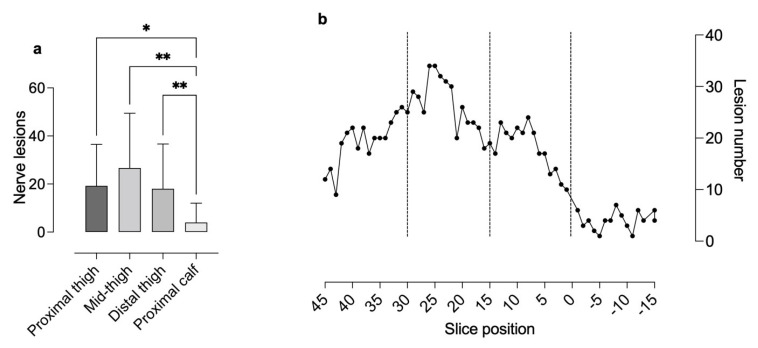
Analyses of peripheral nerve lesion numbers per level (proximal thigh, mid-thigh and distal thigh level and proximal calf) (**a**) and their spatial distribution from proximal to distal (**b**) Analyses of lesion number per level revealed a higher number of microlesions at the thigh levels compared to the calf level. Distribution analysis showed an increase in nerve lesions from the proximal (slice 45 to 30) to the mid-thigh (slice 30 to 15) level and a subsequent decrease towards the distal thigh (slice 15 to 0) and calf (slice 0 to −15) level (Ns non-significant, * *p* < 0.05, ** *p* < 0.01).

The proximal-to-distal lesion count revealed 288 (19.20 ± 4.66) nerve lesions at the proximal thigh level, 400 (26.67 ± 4.98) at the mid-thigh level, 270 (18.00 ± 4.36) at the distal thigh level, and 60 (4.00 ± 1.81) at the proximal calf level. Compared to the proximal calf level, lesion number was significantly higher at the proximal (Bonferroni corrected *p* < 0.05), mid- (Bonferroni corrected *p* < 0.01), and distal thigh level (Bonferroni corrected *p* < 0.01). Between the different thigh levels, no significant differences were found (Figure 2).

**Figure 3:**
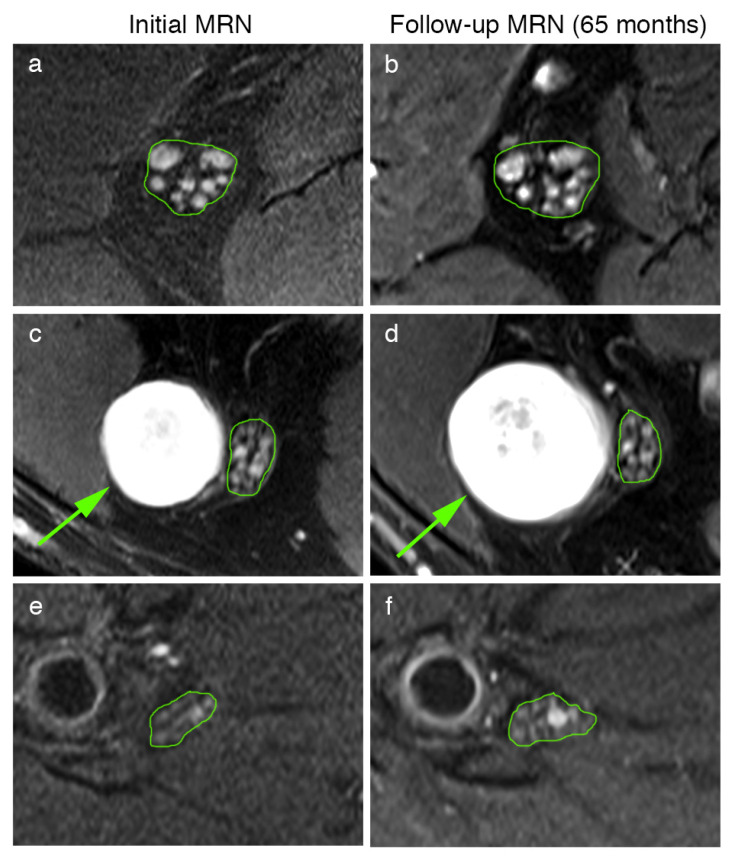
Representative MRN images at different levels of the initial (**a**,**c**,**e**) and follow-up MRN examination (**b**,**d**,**f**) of patient 7’s sciatic nerve at the proximal thigh level (**a**,**b**). Multiple microlesions present at the initial MRN (**a**) show no dynamic change in either their number or their diameter after 65 months (**b**). At the distal thigh level, a solitary macrolesion (arrow in (**c**,**d**)) was observed within the peroneal portion of the sciatic nerve (**c**), which was significantly larger in the follow-up (**d**), whereas adjacent microlesions within the tibial portion remained unchanged (**c**,**d**). In contrast to the high number of microlesions at the thigh levels, these nerve lesions were only occasionally observed at the calf level (**e**,**f**).

Moreover, the peripheral nerve lesion count and lesion diameter were quantitatively assessed for nine schwannomatosis patients in the initial and follow-up examination (Table 1, Figure 3). This analysis revealed that peripheral nerve lesion count did not differ in follow-up examinations for microlesions (total count: 544 vs. 533, 60.33 ± 61.42 vs. 59.22 ± 59.56, *p* = 0.34), intermediate lesions (total count 12 vs. 19, 1.33 ± 4.00 vs. 2.11 ± 5.60, *p* = 0.19), or macrolesions (total count 8 vs. 9, 0.89 ± 2.67 vs. 1.00 ± 3.00, *p* = 0.35). The diameters of the nerve lesions were decreased in follow-up MRN for microlesions (1.08 ± 0.29 mm to 1.03 ± 0.28 mm, *p* < 0.01), did not differ for intermediate lesions (2.67 ± 0.36 mm to 2.56 ± 0.49 mm, *p* = 0.87), and increased for macrolesions (10.63 ± 2.90 mm to 13.72 ± 3.66 mm, *p* < 0.01).

## 4. Discussion

This study examined the spatial distribution and long-term alterations in the peripheral nerve lesions of patients with schwannomatosis and associated SFN. 

A major finding of this study is that peripheral nerve microlesions are the predominant lesion entity in schwannomatosis, distinctly reaching higher lesion counts as opposed to intermediate and macrolesions. This is in accordance with previous studies that described microlesions as the underlying pathostructural correlate of both NF2- and schwannomatosis-associated PNP, with microlesions showing identical histological and imaging features in both syndromes [2,6]. In the past, NF2- PNP was thought to occur as a consequence of a compressive mass effect of schwannomas to adjacent peripheral nerves [7]. Recent studies, however, revealed that multiple intrafascicular tumorlets, also defined as “microlesions” on imaging, seem to be responsible for PNP-related symptoms in the vast majority of NF2 patients [2,7]. Moreover, there seems to be a direct link between the proximal-to-distal accumulation of microlesions and the severity of associated clinical symptoms. In contrast to this finding, a previous study reported that microlesions also occur in the clinically asymptomatic extremities of patients with segmental schwannomatosis [6]. Moreover, microlesions in NF2 are already evident in asymptomatic patients at a very young age to a quite similar extent to NF2-adults with severe PNP [8]. Moreover, most schwannomatosis patients remain asymptomatic during childhood and adolescence and become symptomatic during adulthood. Thus, is seems that in both NF2 and schwannomatosis, there is a direct link between the occurrence and number of these tiny nerve lesions, while secondary processes nearby may be responsible for the development of PNP symptoms. Previous studies described how microlesions can be histologically associated with a pathomorphological finding referred to as “onions bulbs”. These structures develop in areas of repeated de- and remyelinization and are mainly caused by the proliferation of Schwann cells [9,10]. Thus, it could be hypothesized that primary changes to the T2-signal may reflect the pathophysiologically underlying merlin deficiency, but that neuropathic symptoms occur if axonal sprouting and recruiting cannot longer compensate neuronal dysfunction. 

The second major finding of this study is that the number and diameter of peripheral nerve micro- and intermediate lesions are relatively constant or tend to shrink rather than progressing over time, even in long-term follow-up examinations after up to 71 months. By contrast, macrolesions feature a more progressive growth pattern over time but remain stable in number. This is in accordance with a previous study investigating long-term alterations of the peripheral nervous system in NF2, where microlesions were also relatively constant over time [11]. Thus, it could be hypothesized that structural alterations to the peripheral nerves occur very early and show limited instead of linear progress. Possible explanations for this observation could be the reduced growth rate of somatic cells or the lower number of proliferating cells over time [12,13]. 

As pathostructural findings of the peripheral nervous system between NF2 und schwannomatosis show a high degree of similarity, the marked difference in clinical symptoms and PNP type still remains to be explained. While NF2-PNP is predominantly characterized by an axonal loss with an additional, but less pronounced, demyelination and consecutive sensory and motor deficiencies, the most common symptom of schwannomatosis is local, multifocal of diffuse pain. Although the exact pathogenic cause of pain in schwannomatosis remains unclear, previous studies indicate that symptoms are associated with gross schwannoma formation to a lesser extent and are more often consequence of SFN [4,5,14]. This length-dependent neuropathy predominantly affects the unmyelinated C-fibers, causing a reduced IENFD [4]. Although some NF2 patients also show a reduced IENFD, the extent is less pronounced than in schwannomatosis. While chronic pain, the leading symptom of schwannomatosis, is uncommon in NF2 patients, NF2-PNP is characterized by moderate-to-severe sensory deficits and paresis. A previous study found hyperplasia of the DRG as an exclusive imaging and histological feature of NF2 that might serve as a potentially pathostructural correlate of areflexia and sensory loss in NF2-PNP [15]. As peripheral nerve lesions in schwannomatosis are morphologically comparable to those found in patients with NF2, a further discriminatory pattern might exist. 

As a third major finding, this study revealed a markedly different spatial distribution of peripheral nerve lesions in schwannomatosis. While for NF2, a cumulative lesion pattern with an increasing number of peripheral nerve lesions from proximal to distal was described [2], in schwannomatosis, the maximum extent of nerve lesions is present at the mid-thigh level, with a marked decrease towards the distal site. This lesion pattern with a decreasing gradient from the proximal to the distal regions could also be observed in various other types of SFN, such as Fabry neuropathy or diabetic neuropathy, the most prevalent human neuropathy. A pervious study investigating the distribution of peripheral nerve lesions in patients with type 1 or 2 diabetes and associated neuropathy described a clear proximal-to-distal gradient of nerve lesions, with a predominance of lesions at the thigh level [16]. In this way, the gradient of nerve lesion distribution differs markedly from the longitudinal gradient of neuropathic symptoms, which typically follow the opposite (distal-to-proximal) direction. Thus, SFN in schwannomatosis shares not only shares, but also imaging features with other types of SFN. Several possible pathomechanisms for the development of neuropathic pain in schwannomatosis, such as the increased production of inflammatory cytokines or the impairment of neurotrophic factors, have been discussed so far [17,18,19,20]. A further reasonable explanation is that structural alterations in the neuronal microcirculation and metabolic nutrition begin at proximal sites, accumulate from proximal to distal regions, and eventually lead to length-dependent fiber loss at more distal levels. The reason why nerve segments at the thigh level are more susceptible to microstructural alterations is still unknown. Altogether, the accumulation of microstructural nerve alterations at the thigh level may precede and possibly trigger distal fiber loss in a length-dependent manner, in which the distal fibers tend to lose their structural integrity and function first.

A limitation of this study is that inter- and intrareader analyses were not performed to achieve a direct 1:1 nerve lesion correlation as only one radiologist reviewed the MRN data. However, previous studies investigated the intra- and interrater agreement over the detection of peripheral nerve lesions in MRN and found a high agreement [11,21].

## 5. Conclusions

Microlesions represent the predominant type of peripheral nerve lesion in schwannomatosis and show a relatively consistent pattern in long-term follow-up. In contrast to the distally predominant distribution of nerve lesions in NF2, the lesion pattern in schwannomatosis peaks at the mid-thigh level, with a subsequent decrease in the quantity of nerve lesions in the distal nerve segments. This pattern therefore shares general features with other types of SFN. 

## Figures and Tables

**Table 1:** Patient demographics and clinical data.

	Gender and Age in Years (Initial)	Mutation	MRN Follow-Up (Months)	Distribution of Peripheral Nerve Lesions	Pain	Sensibility	Ankle Jerk Reflex	PNP	New Clinical Symptoms (Initial—Follow-Up)	Sciatic, Tibial and Peroneal Nerve Lesions (Initial)	Sciatic, Tibial, and Peroneal Nerve Lesions (Follow-Up)	IENFD	<5% Norm/Decade	IENFD Decrease
1	F, 36	VUS c.1288C>T; p.His 430 Tyr	53	Pelvic: 0 Proximal thigh: 12 Mid-thigh: 24 Distal thigh: 2 Calf: 0	Yes	Normal	Normal	None	None	Micro: 38 Intermediate: 0 Macro: 0	Micro: 37 Intermediate: 0 Macro: 0	1.5	7.1	−78.9%
2	F, 38	Not determined	Not performed	Pelvic: 0 Proximal thigh: 31 Mid-thigh: 27 Distal thigh: 2 Calf: 0	Yes	Decreased post op	Decreased	None	n.a.	Micro: 60 Intermediate: 0 Macro: 0	n.a.	2	7.1	−71.8%
3	F, 57	Not detected LZTR1, SMARCB1, NF2	71	Pelvic: 0 Proximal thigh: 1 Mid-thigh: 1 Distal thigh: 8 Calf: 1	Yes	Decreased post op	Normal	None	Painful plexus lesion	Micro: 11 Intermediate: 0 Macro: 0	Micro: 10 Intermediate: 0 Macro: 0	1.2	3.2	−62.5%
4	M, 55	Not detected LZTR1, SMARCB1, NF2	39	Pelvic: 0 Proximal thigh: 13 Mid-thigh: 22 Distal thigh: 12 Calf: 2	Yes	Normal	Normal	None	None	Micro: 49 Intermediate: 0 Macro: 0	Micro: 49 Intermediate: 1 Macro: 0	1.9	3.5	−45.7%
5	M, 42	Not detected LZTR1, SMARCB1, NF2	Not performed	Pelvic: 0 Proximal thigh: 26 Mid-thigh: 16 Distal thigh: 0 Calf: 1	Yes	Normal	Normal	None	n.a.	Micro: 43 Intermediate: 0 Macro: 0	n.a.	n.a.	n.a.	n.a.
6	M, 45	Not determined	Not performed	Pelvic: 0 Proximal thigh: 56 Mid-thigh: 58 Distal thigh: 23 Calf: 0	Yes	Decreased	Decreased	Yes	n.a.	Micro: 94 Intermediate: 48 Macro: 4	n.a.	1.0	4.4	−77.3%
7	M, 52	Not detected LZTR1, SMARCB1, NF2	65	Pelvic: 2 Proximal thigh: 48 Mid-thigh: 86 Distal thigh: 75 Calf: 25	Yes	Decreased	Decreased	Yes	Foot drop	Micro: 214 Intermediate: 12 Macro: 8	Micro: 206 Intermediate: 17 Macro: 9	1.7	3.5	−51.4%
8	M, 56	Not detected LZTR1, SMARCB1, NF2	Not performed	Pelvic: 0 Proximal thigh: 18 Mid-thigh: 31 Distal thigh: 29 Calf: 22	Yes	Decreased post op	Normal	Not performed	n.a.	Micro: 95 Intermediate: 3 Macro: 2	n.a.	n.a.	n.a.	n.a.
9	M, 40	LZTR1 978-985delCAGCTCCG	70	Pelvic: 0 Proximal thigh: 13 Mid-thigh: 3 Distal thigh: 14 Calf: 0	Yes	Normal	Normal	None	None	Micro: 30 Intermediate: 0 Macro: 0	Micro: 30 Intermediate: 0 Macro: 0	2.0	4.4	−54.5%
10	F, 52	Not detected LZTR1, SMARCB1, NF2	Not performed	Pelvic: 1 Proximal thigh: 6 Mid-thigh: 30 Distal thigh: 23 Calf: 3	Yes	Normal	Normal	None	n.a.	Micro: 62 Intermediate: 0 Macro: 0	n.a.	n.a.	n.a.	
11	M, 45	Not detected LZTR1, SMARCB1, NF2	60	Pelvic: 3 Proximal thigh: 14 Mid-thigh: 23 Distal thigh: 25 Calf: 0	Yes	Normal	Normal	None	None	Micro: 62 Intermediate: 0 Macro: 0	Micro: 64 Intermediate: 0 Macro: 0	2.1	4.4	−52.2%
12	M, 62	Not detected LZTR1, SMARCB1, NF2	65	Pelvic: 2 Proximal thigh: 8 Mid-thigh: 49 Distal thigh: 21 Calf: 0	Yes	Decreased post op	Normal	None	None	Micro: 78 Intermediate: 0 Macro: 2	Micro: 81 Intermediate: 1 Macro: 0	1.2	2.8	−57.1%
13	M, 55	Not detected LZTR1, SMARCB1, NF2	46	Pelvic: 0 Proximal thigh: 19 Mid-thigh: 13 Distal thigh: 11 Calf: 3	Yes	Decreased	Absent	Yes	None	Micro: 46 Intermediate: 0 Macro: 0	Micro: 44 Intermediate: 0 Macro: 0	3.0	3.5	−14.2%
14	F, 54	c.1312G>T;p.Glu438	42	Pelvic: 0 Proximal thigh: 6 Mid-thigh: 9 Distal thigh: 0 Calf: 1	Yes	Normal	Normal	None	None	Micro: 16 Intermediate: 0 Macro: 0	Micro: 12 Intermediate: 0 Macro: 0	4.3	4.3	0%
15	F, 56	Not detected LZTR1, SMARCB1, NF2	Not performed	Pelvic: 2 Proximal thigh: 8 Mid-thigh: 8 Distal thigh: 25 Calf: 2	No	Normal	Normal	None	n.a.	Micro: 43 Intermediate: 0 Macro: 0	n.a.	n.a.	n.a.	

## Data Availability

The data that support the findings of this study are available on request from the corresponding author. The data are not publicly available due to restrictions, as they contain information that could compromise the privacy of the research participants.

## References

[1] Farschtschi S., Mautner V.F., McLean A.C.L., Schulz A., Friedrich R.E., Rosahl S.K. (2020). The Neurofibromatoses. Dtsch. Arztebl. Int..

[2] Baumer P., Mautner V.F., Baumer T., Schuhmann M.U., Tatagiba M., Heiland S., Kaestel T., Bendszus M., Pham M. (2013). Accumulation of non-compressive fascicular lesions underlies NF2 polyneuropathy. J. Neurol..

[3] Hagel C., Lindenau M., Lamszus K., Kluwe L., Stavrou D., Mautner V.F. (2002). Polyneuropathy in neurofibromatosis 2: Clinical findings, molecular genetics and neuropathological alterations in sural nerve biopsy specimens. Acta Neuropathol..

[4] Farschtschi S.C., Mainka T., Glatzel M., Hannekum A.L., Hauck M., Gelderblom M., Hagel C., Friedrich R.E., Schuhmann M.U., Schulz A. (2020). C-Fiber Loss as a Possible Cause of Neuropathic Pain in Schwannomatosis. Int. J. Mol. Sci..

[5] Farschtschi S.C., Kluwe L., Schon G., Friedrich R.E., Matschke J., Glatzel M., Weis J., Hagel C., Mautner V.F. (2020). Distinctive low epidermal nerve fiber density in schwannomatosis patients provides a major parameter for diagnosis and differential diagnosis. Brain Pathol..

[6] Farschtschi S., Mautner V.F., Pham M., Nguyen R., Kehrer-Sawatzki H., Hutter S., Friedrich R.E., Schulz A., Morrison H., Jones D.T. (2016). Multifocal nerve lesions and LZTR1 germline mutations in segmental schwannomatosis. Ann. Neurol..

[7] Sperfeld A.D., Hein C., Schroder J.M., Ludolph A.C., Hanemann C.O. (2002). Occurrence and characterization of peripheral nerve involvement in neurofibromatosis type 2. Brain.

[8] Godel T., Baumer P., Farschtschi S., Gugel I., Kronlage M., Hofstadler B., Heiland S., Gelderblom M., Bendszus M., Mautner V.F. (2019). Peripheral nervous system alterations in infant and adult neurofibromatosis type 2. Neurology.

[9] Gehlhausen J.R., Park S.J., Hickox A.E., Shew M., Staser K., Rhodes S.D., Menon K., Lajiness J.D., Mwanthi M., Yang X. (2015). A murine model of neurofibromatosis type 2 that accurately phenocopies human schwannoma formation. Hum. Mol. Genet..

[10] Onishi A., Nada O. (1972). Ultrastructure of the onion bulb-like lamellated structure observed in the sural nerve in a case of von Recklinghausen’s disease. Acta Neuropathol..

[11] Godel T., Baumer P., Farschtschi S., Puschel K., Hofstadler B., Heiland S., Gelderblom M., Bendszus M., Hagel C., Mautner V.F. (2021). Long-term Follow-up and Histological Correlation of Peripheral Nervous System Alterations in Neurofibromatosis Type 2. Clin. Neuroradiol..

[12] Baser M.E., Makariou E.V., Parry D.M. (2002). Predictors of vestibular schwannoma growth in patients with neurofibromatosis Type 2. J. Neurosurg..

[13] Baser M.E., Mautner V.F., Parry D.M., Evans D.G. (2005). Methodological issues in longitudinal studies: Vestibular schwannoma growth rates in neurofibromatosis 2. J. Med. Genet..

[14] Merker V.L., Esparza S., Smith M.J., Stemmer-Rachamimov A., Plotkin S.R. (2012). Clinical features of schwannomatosis: A retrospective analysis of 87 patients. Oncologist.

[15] Godel T., Mautner V.F., Farschtschi S., Pham M., Schwarz D., Kronlage M., Gugel I., Heiland S., Bendszus M., Baumer P. (2018). Dorsal root ganglia volume differentiates schwannomatosis and neurofibromatosis 2. Ann. Neurol..

[16] Pham M., Oikonomou D., Hornung B., Weiler M., Heiland S., Baumer P., Kollmer J., Nawroth P.P., Bendszus M. (2015). Magnetic resonance neurography detects diabetic neuropathy early and with Proximal Predominance. Ann. Neurol..

[17] Wagner R., Myers R.R. (1996). Schwann cells produce tumor necrosis factor alpha: Expression in injured and non-injured nerves. Neuroscience.

[18] Abbadie C., Lindia J.A., Cumiskey A.M., Peterson L.B., Mudgett J.S., Bayne E.K., DeMartino J.A., MacIntyre D.E., Forrest M.J. (2003). Impaired neuropathic pain responses in mice lacking the chemokine receptor CCR2. Proc. Natl. Acad. Sci. USA.

[19] Naruse K. (2019). Schwann Cells as Crucial Players in Diabetic Neuropathy. Adv. Exp. Med. Biol..

[20] Tyner T.R., Parks N., Faria S., Simons M., Stapp B., Curtis B., Sian K., Yamaguchi K.T. (2007). Effects of collagen nerve guide on neuroma formation and neuropathic pain in a rat model. Am. J. Surg..

[21] Preisner F., Behnisch R., Foesleitner O., Schwarz D., Wehrstein M., Meredig H., Friedmann-Bette B., Heiland S., Bendszus M., Kronlage M. (2021). Reliability and reproducibility of sciatic nerve magnetization transfer imaging and T2 relaxometry. Eur. Radiol..

